# Thermally Assisted Photocatalytic Dehydrogenation of Methanol in Continuous Steady‐State Operation

**DOI:** 10.1002/advs.76842

**Published:** 2026-07-27

**Authors:** Florian Stubenrauch, Phillip Nathrath, Markus Schörner, Yazan Mahayni, Birk Fritsch, Andreas Hutzler, Peter Wasserscheid, Andreas Bösmann, Patrick Schühle

**Affiliations:** ^1^ Helmholtz‐Institut Erlangen‐Nürnberg for Renewable Energy (IEK11) Forschungszentrum Jülich GmbH Erlangen Germany; ^2^ Institute of Chemical Reaction Engineering Friedrich‐Alexander‐Universität Erlangen‐Nürnberg Erlangen Germany; ^3^ Institute For a Sustainable Hydrogen Economy (IHE) Forschungszentrum Jülich GmbH Jülich Germany

**Keywords:** dehydrogenation, formaldehyde, hydrogen, methanol, photocatalysis, platinum, titanium dioxide

## Abstract

The direct dehydrogenation of methanol to produce formaldehyde has long been considered a dream reaction, because it promises higher product value, lower safety hazards, and lower CO_2_ emissions compared to state‐of‐the‐art methanol oxidation. However, thermodynamic equilibrium constraints prevent competitive yields under purely thermocatalytic conditions. This study presents thermally assisted photocatalytic dehydrogenation of methanol as a method enabling formaldehyde yields far beyond the limits of pure thermocatalysis. In continuous, stable operation over 70 h, formaldehyde selectivity of 95% and methanol conversions up to 60 times above thermodynamic equilibrium are achieved. The setup consists of a flow reactor with a catalytically active Pt/TiO_2_‐coated plate, which is irradiated with UV light from the top, heated from the backside, and continuously fed with methanol. Variation of temperature (78–120°C), residence time (6.1–23.5 s), and irradiation intensity (118.1–255.8 mW cm^−^^2^) shows that high values of all three parameters are required to maximize formaldehyde and hydrogen yields. Continuous co‐dosing of oxygen at very low concentrations (≤1 mol%) significantly increases conversion and shifts selectivity toward methyl formate. Our findings suggest that oxygen microdosing allows replenishment of surface oxygen sites on TiO_2_, which act as key adsorption centers in the thermally assisted photocatalytic process.

## Graphical abstract

Thermally assisted photocatalytic methanol dehydrogenation on Pt/TiO_2_ enables formaldehyde and hydrogen production far beyond thermodynamic limits. Continuous operation delivers up to 95% selectivity over 70 h, and oxygen microdosing strongly boosts conversion by regenerating surface oxygen sites.

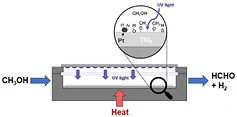

## 1 Introduction

Formaldehyde (HCHO) is an important chemical for the industrial production of resins and polymers. In addition, HCHO has gained interest for the production of polyoxymethylene ethers (OME), which represent interesting fuels or fuel additives for cleaner and more sustainable combustion processes [1]. HCHO is conventionally produced by selective oxidation of methanol (MeOH) over Ag‐ or MoFe‐catalysts, e.g. using the so‐called FormOx process [2].

(1) $$CH_{3}\mathrm{OH}+0.5\;O_{2}\;↔\;\mathrm{HCHO}+H_{2}O\to \Delta H_{R}=-159\;\mathrm{kJmo}l^{-1}$$

Despites its commercial operation over decades and continuously attempts for its improvement, the selective oxidation pathway still exhibits several drawbacks: (i) Competing reaction pathways form CO and CO_2_ as undesired byproducts; (ii) Handling the potentially explosive mixtures formed by the organic feed and the oxidant—either reactant ratios are chosen to stay outside the explosion range or explosion‐proof components and the respective plant designs and security measures are used—both options produce additional cost for the process; (iii) The formation of the stoichiometric byproduct water (see Equation 1) causes tedious purification processes to produce dry HCHO.

All these drawbacks could be solved in one stroke by applying direct dehydrogenation of methanol to produce formaldehyde (see Equation 2). This alternative pathway would additionally enable the production of valuable hydrogen as a stoichiometric byproduct. After separation, this hydrogen could either be recycled to the methanol production unit, used for other processes or electrified in a hydrogen fuel cell. Coupled with hydrogen production, the absence of safety hazards and the significantly reduced probability of CO_2_ formation render direct methanol dehydrogenation a highly desirable reaction.

(2) $$CH_{3}\mathrm{OH}↔\mathrm{HCHO}+H_{2}\to \Delta H_{R}=+84\;\mathrm{kJ}\;\mathrm{mo}l^{-1}$$

However, the thermodynamic equilibrium of the direct MeOH dehydrogenation is unfavorable, making only very limited conversions reachable at reasonable reaction temperatures (simulated reaction equilibrium data are shown in Figure S1) [3, 4, 5]. This shows the need for non‐thermal activation to overcome these limits for reaching practically relevant HCHO yields. By using irradiation energy (either solar or artificial) in heterogeneous photocatalysis, excited electronic states of the catalyst can be generated, which enable otherwise endergonic reactions or help to overcome activation energies in exergonic reactions [6, 7, 8, 9]. Since the pioneering work of Fujishima and Honda in 1972 [10], the most intensively studied photocatalyst is titania (TiO_2_) in its crystal phases rutile or anatase. Such titania is cheap and widely commercially available (e.g., Aeroxide P25).

The photocatalytic dehydrogenation of MeOH to formaldehyde and hydrogen has been investigated by various groups in the past. These studies focused on fundamental aspects of the photocatalyst design and its mechanistic understanding [11, 12, 13, 14, 15, 16]. The group of Heiz, for example, recently postulated a mechanism for the dehydrogenation of alcohols in gas‐phase reactions based on studies in ultra‐high vacuum (UHV) for Pt deposited on rutile (110) [17, 18]. The proposed mechanism includes thermo‐ and photo‐induced steps (see Equations (3), (4), (5), (6), (7)), with O_sb_ representing surface oxygen atoms, including bridge‐bonded oxygen and oxygen adatoms.

(3) $$\begin{array}{ccc} {\left({*}_{\mathrm{Ti}O_{2}}\right)}^{-} & + & CH_{3}\mathrm{OH}+O_{sb}\to {\left(CH_{3}O-{*}_{\mathrm{Ti}O_{2}}\right)}^{-}\\ & & +HO_{sb}\to \left(\mathrm{Thermo}-\mathrm{induced}\right) \end{array}$$

(4) $$\begin{array}{ccc} {\left(CH_{3}O-{*}_{\mathrm{Ti}O_{2}}\right)}^{-} & + & h^{+}+O_{sb}\to \mathrm{HCHO}-{*}_{\mathrm{Ti}O_{2}}\\ & & +HO_{sb}\to \left(\mathrm{Photo}-\mathrm{induced}\right) \end{array}$$

(5) $$\mathrm{HCHO}-{*}_{\mathrm{Ti}O_{2}\;}\to \mathrm{HCH}O_{\left(g\right)}+{*}_{\mathrm{Ti}O_{2}\;}\to \left(\mathrm{Thermo}-\mathrm{induced}\right)$$

(6) $$2HO_{sb}\overset{\left[\mathrm{Pt}\right]}{\to }H_{2}+2O_{sb}\to \left(\mathrm{Thermo}-\mathrm{induced}\right)$$

(7) $${*}_{\mathrm{Ti}O_{2}}+e^{-}\to {\left({*}_{\mathrm{Ti}O_{2}}\right)}^{-}\to \left(\mathrm{Photo}-\mathrm{induced}\right)$$

1. In a first step, MeOH adsorbs dissociatively on a negatively charged defect Ti‐site ${\left({*}_{\mathrm{Ti}O_{2}}\right)}^{-}$ forming a methoxy species and a formally neutral hydrogen atom bound to a surface oxygen O_sb_. The cleavage of the alcohol group is thermally induced [19, 20, 21, 22].
2. The photoactive methoxy reacts under illumination with a photogenerated hole, resulting in a homolytic cleavage of a C─H bond [22]. As a result, formaldehyde bound to Ti and another hydrogen atom bound to surface oxygen are formed. Note that the backwards reaction of adsorbed HCHO with surface hydroxyls is described as thermal recombination in the literature [23].
3. Formaldehyde desorbs in another thermal step, a step that quickly proceeds even at room temperature [24].
4. The produced hydrogen atoms remain bound to the surface, but are mobile and diffuse to the Pt particle [25, 26]. Here, the H─H bond is formed by recombination, and H_2_ desorbs. The sole purpose of Pt is to act as a recombination site for hydrogen, as shown by Joo et al. [27].
5. The photoelectron finally restores the negative charge at the surface Ti [17, 18].

A similar reaction mechanism was postulated by Setvin et al. for MeOH on anatase (the main part of P25) [28], based on experimental STM, XPS, and TPD data and theoretical DFT calculations [29]. As no noble metal was present in their study, molecular hydrogen was not formed. These studies furthermore reveal that the thermal dissociation of adsorbed MeOH on anatase (Equation 3) is significantly enhanced at higher coverages, while at low coverages terminal OH^−^ groups or co‐adsorbed O_2_ are required on the surface to facilitate the methoxy formation [29, 30].

Other recent studies by Zhang et al. [19] as well as Chai [15] suggest that the first step (Equation 3) does not require a negatively charged Ti^3+^‐defect site but can also proceed at Ti^4+^ sites. In this case, the cleavage of the alcohol group results in a negatively charged methoxy group, bound to the Ti‐site, and a proton, bound to the lattice oxygen. Afterwards, the methoxy reacts with the photogenerated hole to form a radical, and via a subsequent homolytic cleavage, formaldehyde and a hydrogen atom are bound to lattice oxygen. The photoelectron reduces the proton and forms a second hydrogen atom. The two hydrogen atoms diffuse to the metal surface and can afterwards desorb associatively as molecular hydrogen. While Zhang et al. [19] postulated via DFT+U calculations that the overall mechanism happens via a two‐photon process, Walenta et al. [18] and Katsiev et al. [31] proposed that only one photon is involved. Although differences in some mechanistic details remain, the following general reaction sequence has been reported across all of these studies for the metal supported, photocatalytic dehydrogenation of MeOH: (i) thermally induced dissociation of MeOH resulting in methoxy (bound to Ti) and hydrogen species (bound to surface oxygen); (ii) photo‐induced homolytic cleavage of a C─H‐bond; (iii) thermal desorption of formaldehyde; (iv) recombination of molecular hydrogen on metal surface, followed by (v) thermo‐induced hydrogen desorption.

Besides formaldehyde, methyl formate (MF) can be formed as a secondary product. MF itself is a valuable chemical building block and energy carrier [32, 33, 34, 35]. In the context of the described mechanism, MF is formed via various coupling reactions between formaldehyde and methoxy species [36, 37, 38, 39, 40, 41]. Phillips et al. and Zhang et al. describe the generation of a transient formyl species (Equation 8), that reacts with a methoxy species to MF (Equation 9) [41, 42]. Another pathway to MF is the dimerization of formaldehyde via Tischenko‐type reactions (Equation 10) [40, 43]. For this thermal reaction step, both acidic (e.g., Ti^4+^) and basic (OH^−^ or O^2−^) functionalities are required in close proximity on the catalyst [44, 45]. Furthermore, HCHO readily reacts with MeOH via semi‐acetalization to hemiformal (H_3_COCH_2_OH, HeFal) in a thermally controlled equilibrium reaction (Equation 11). HeFal can act as an intermediate and dehydrogenates to MF [43]. Another thermal reaction that might occur at high surface coverages is the recombination of two hydroxyls to water (Equation 12), creating a bridging oxygen vacancy on a reduced surface or an oxygen adatom on an oxidized surface [22, 46, 47].

As described earlier, surface‐bound oxygen (O_sb_) plays a crucial role in the photocatalytic methanol dehydrogenation on TiO_2_ [22, 48, 49]. Consequently, a loss of O_sb_ can be responsible for deactivation of the photocatalyst. From adsorption studies, it is known that oxygen scrambling, i.e., exchange of O_sb_ of TiO_2_ with adsorbed molecules, is possible, suggesting that O_sb_ species are not static but are dynamically involved in various reactions relevant to the overall mechanism of MeOH dehydrogenation (see Equations (3), (4), (6), (8) and 12) [50, 51, 52, 53].

(8) $$\mathrm{HCHO}-{*}_{\mathrm{Ti}O_{2}}+O_{sb}\;\overset{\mathrm{hv}}{\to }\;\mathrm{HCO}-{*}_{\mathrm{Ti}O_{2}}+HO_{sb}$$

(9) $$\mathrm{HCO}-{*}_{\mathrm{Ti}O_{2}}+\;CH_{3}O-{*}_{\mathrm{Ti}O_{2}}\;\to \mathrm{HCOOC}H_{3}↑+2\;{*}_{\mathrm{Ti}O_{2}}$$

(10) $$2\;\mathrm{HCHO}\;\to \;\mathrm{HCOOC}H_{3}$$

(11) $$CH_{3}\mathrm{OH}+\mathrm{HCHO}\;↔H_{3}\mathrm{COC}H_{2}\mathrm{OH}$$

(12) $$2\;HO_{sb}\;\to \;H_{2}O_{\left(g\right)}+O_{sb}$$

Despite these solid physicochemical and surface science insights into the photocatalytic MeOH dehydrogenation, studies that have further developed this reaction towards a technical use are scarce to date [11, 14, 54, 55]. The vast majority of studies have been conducted in batch reactors and primarily focus on catalyst development and hydrogen evolution rates rather than reporting process‐level methanol conversions and formaldehyde yields [56, 57]. Studies addressing continuous photocatalytic methanol dehydrogenation remain scarce. Jiao et al. reported methanol conversions of up to 6.7% in a continuously operated membrane reactor [14], while Hussein et al. recently demonstrated continuous operation of an Au/TiO_2_ catalyst but did not report methanol conversions or formaldehyde yields [58]. This highlights the need for continuous‐flow studies that enable the assessment of process performance using conversion and product yield as key metrics. To the best of our knowledge, photocatalytic methanol dehydrogenation has not been substantially studied in a continuous‐flow system under steady‐state conditions for determining productivity and selectivity data under optimal operating parameters and for assessing catalyst stability—all of which are key performance indicators for the practical use of this technology.

In this work, we investigate the thermally assisted photocatalytic MeOH dehydrogenation in a continuous flow plate reactor applying a Pt/TiO_2_ catalyst. Our study examines the influence of photocatalyst pretreatment, reaction temperature, residence time, and irradiation intensity on the HCHO and H_2_ yields. Furthermore, we investigate the effect of adding trace amounts of oxygen to enhance catalyst activity by maintaining oxidized surface sites, whilst minimizing the oxidation of hydrogen to water. Our reaction engineering approach enables stable product yields that exceed the thermodynamic equilibrium achievable with purely thermal activation, which is an essential prerequisite for the technical implementation of this highly promising reaction.

## 2 Results and Discussion

### 2.1 Catalyst Characterization

**FIGURE 1:**
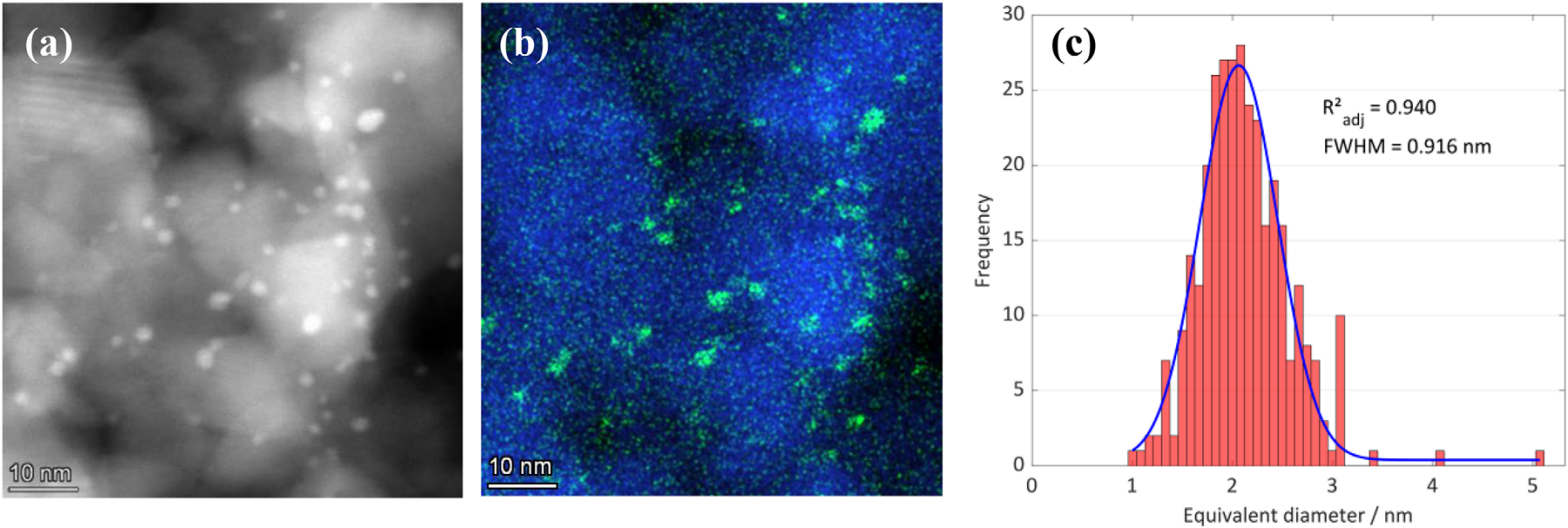
(a) HAADF‐STEM image of the used Pt/TiO_2_ catalyst; (b) EDX spectrum image (blue = Ti, green = Pt); (c) particle size distribution of the Pt/TiO_2_ catalyst applied in this study: size histogram with Gaussian fitted curve.

The prepared photocatalyst was characterized in its powdered form before spray coating via HAADF‐STEM and EDXS. A representative image is shown in Figure 1a. The platinum particles are visible as brighter areas (mass contrast) on the surface of the TiO_2_ particles (∼40 nm diameter) as confirmed by the EDX spectrum image (Figure 1b). Their shape appears to be roughly spherical or elliptical. From different images, a total of 327 particles were counted for size analysis. From the areas measured, the diameter of volume‐equivalent spheres was calculated. The resulting histogram with a Gaussian‐fitted distribution (R^2^_adj._ = 0.94, fitting equation, parameters and respective errors in Table S1) can be seen in Figure 1c. Our catalyst shows a peak Pt‐particle size of 2.10 nm with a reasonably narrow distribution (FWHM = 0.92 nm). The total platinum content was measured by ICP‐AES to be 0.39 wt.%. Given that the coating procedure operates at mild temperatures (≤100°C), any morphological changes or thermal sintering of the catalyst during preparation of the catalyst‐coated plates is avoided.

### 2.2 Catalyst Activation and Deactivation

**FIGURE 2:**
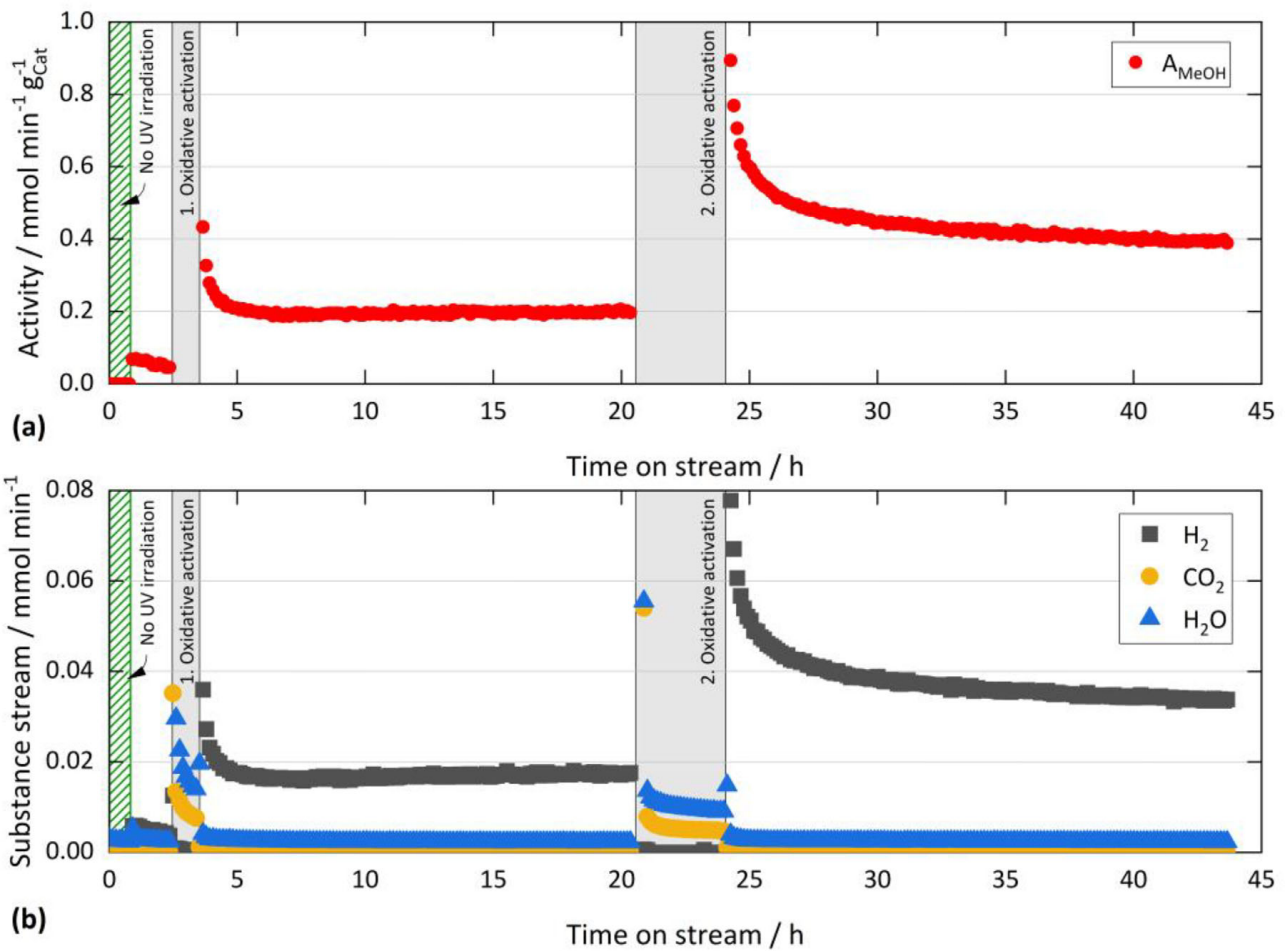
Role of oxidative catalyst pretreatment (time intervals in grey) in the photocatalytic MeOH dehydrogenation. (a) Activity and (b) substance streams for H_2_, CO_2_ and H_2_O over time‐on‐stream. Activation conditions: ‘standard’, 3 vol.% O_2_ in N_2_ at T = 78°C and E = 186.8 mW cm^−2^; Reaction conditions: 10 vol.% MeOH in N_2_, E = 186.8 mW cm^−2^, T = 78°C, τ = 11.8 s, m_Cat_ = 0.1006 g.

Different pretreatment procedures were tested to activate the photocatalyst in the most effective way. While a reductive pretreatment (3 vol.% H_2_ at 100°C dark/irradiated), which we tested with the intention to reduce oxidized Pt‐centers from catalyst preparation, had no beneficial effect on the photocatalytic activity (see Figure S3), an oxidative pretreatment proved much more effective. Figure 2 shows the photocatalytic performance of a freshly prepared catalyst‐coated plate, initially without any pretreatment, at 78°C. After switching on the irradiation (186.8 mW cm^−2^ irradiation intensity) after an initial dark phase of 0–0.9 h time‐on‐stream, a low activity of 0.06 mmol min^−1^ g^−1^_Cat_ was observed.

An oxidizing pretreatment was then applied after 2.5 h time‐on‐stream for one hour using 3 vol.% O_2_ in N_2_ as oxidant. This measure was taken to oxidize blocking surface organic residues from the synthesis and to replenish surface oxygen species (O_sb_) in the TiO_2_. In contrast to the reductive pretreatment, the oxidative treatment had a strongly positive effect on activity, which was also reproduced several times with the same catalyst sample (see Figure S4 for details). In the experiment shown in Figure 2, such oxidative treatment was applied a second time after 21 h time‐on‐stream, this time for a duration of 3 h. Every time, the oxidative treatment procedure led to a significant increase in activity for the subsequent hours of operation. Following the adjustment to the reaction conditions at TOS = 3.6 h, the activity rose to over 0.4 mmol min^−1^ g^−1^_Cat_, followed by a fast decay of activity (TOS = 3.6 – 4.8 h), before reaching a stable operation point at 0.2 mmol min^−1^ g^−1^_Cat_. The second 3 h oxidative treatment led to an even stronger increase in activity. Here, an initial value of approx. 0.9 mmol min^−1^ g^−1^_Cat_ was observed, followed by the decay and a subsequent stable operation point at around 0.4 mmol min^−1^ g^−1^_Cat_.

The molar quantities of H_2_, H_2_O and CO_2_ formed during the activation and reaction phases are illustrated in Figure 2b. While water and CO_2_ contents were very low or absent during the dehydrogenation periods, higher water and CO_2_ quantities were measured during both oxidation periods (see Figure 2b). The detected ratio of H_2_O to CO_2_ was approximately 1.9 during both activating oxidative treatments (see also Figure S5). The formation of both compounds is high at the beginning of the activation period and drops exponentially afterwards, staying at a lower stagnant level throughout the rest of the activation period. This suggests that surface‐bound hydrocarbon species (e.g., formyl species) are oxidized during the activation period. The liberation of the surface sites may therefore contribute to subsequent reactivation. In the first activation, this might be attributed to a residue from the catalyst preparation process or from the short test period under dark conditions, as indicated by the fact that the activity of the freshly prepared catalyst is low from the start. In the case of the reactivation of a previously used catalyst, the hydrocarbon species probably stems from adsorbed feed species or products, as discussed later. The comparatively low activity of 0.2 mmol min^−1^ g^−1^_Cat_ after the first oxidative activation in Figure 2 is likely related to the high amount of adsorbed surface species present after the catalyst had undergone a dark phase and a reaction phase without prior activation during the first 3 h TOS. In contrast, in Figure S4 the oxidative treatment was applied directly to the pristine catalyst surface, so that 1 h of oxidation was sufficient to reach activities of around 0.4 mmol min^−1^ g^−1^_Cat_ in the first reactivation. Interestingly, Katsiev et al. also observed the need to activate the catalyst surface in the dehydrogenation of ethanol over Au/TiO_2_ (110) [31]. In their case, an activation was carried out by exposing the surface covered with ethanol to UV irradiation only, which did not work sufficiently in our case (see Figure S3 for details).

From a comparison of the H_2_ and the H_2_O formation during reaction (see Figure 2b), it can be concluded whether formaldehyde is produced via methanol dehydrogenation (H_2_ formation as stoichiometric coupling product) or via the methanol oxidation pathway (H_2_O formation as stoichiometric coupling product). A low level of water contamination (approx. 2.4 µmol min^−1^) can be observed under dark conditions (0–0.9 h, green hatching), which represents an impurity in the MeOH feed. When switching from the oxidative activation phase back to reaction conditions, the water formation abruptly drops to the feed contamination level and H_2_ is formed instead, following the trend of catalyst activity (compare Figure 2a). This unambiguously demonstrates that MeOH dehydrogenation and not oxidation is the dominant pathway for formaldehyde formation under the present oxygen‐free reaction conditions. Consequently, the higher activity observed after oxidative treatment is likely associated with an altered catalyst state, i.e., the formation of additional O_sb_ during the oxidative treatment that promotes C─H cleavage (Equation (3), (4), and 8).

After the second activation phase, a slight but steady loss in catalyst activity is observed. Because the product concentration in the reactor is much higher after the second activation (see Figure 2b), a deactivation mechanism that involves the formed reaction products is likely. HCHO is known to react readily with itself (i.e., formation of 1,3,5‐trioxane or paraformaldehyde). Moreover, it also reacts with MeOH forming hemiformal, CH_3_OCH_2_OH. The latter can grow to higher chain length by further addition of HCHO. All these heavier products have high melting points and low vapor pressures, so that catalyst site‐blocking by carbonaceous deposits cannot be excluded.

To further elucidate this potential deactivation pathway, we compared TGA‐MS measurements of a used and a freshly prepared Pt/TiO_2_ catalyst. This comparison revealed that carbonaceous species are adsorbed on the catalyst surface after long‐term operation (see Figure S6 for details). Although the exact composition of these deposits is unclear, the oxidation temperature range of 180–380°C indicates strongly bound formate species or adsorbed polymeric structures (i.e., para‐formaldehyde or polyoxymethylene species) [50, 59]. Graphitic carbon, which is characterized by oxidation temperatures above 500°C, was not observed. Noteworthy is that the purely thermal oxidation in the TGA experiment requires at least 180°C to observe any conversion, while the use of UV‐light enables efficient oxidation on the catalyst at temperatures as low as 78°C. Similar effects have been described by Caravaca et al. [60].

Another possible catalyst deactivation mechanism is the poisoning of the Pt nanoparticles with CO formed by thermal decomposition of MeOH [24, 61]. However, no CO was observed within the measurable range of micro‐GC in the reactor exhaust gas. This is also in good agreement with the observations made by Caravaca et al. in their study of methanol photoreforming [60]. For both deactivation mechanisms (heavy formation, CO poisoning), a reactivation by treatment with O_2_ under UV‐irradiation and with formation of CO_2_ seems very reasonable. The equally observed H_2_O formation during the activation treatment indicates, however, a higher proportion of hydrocarbon oxidation vs. CO oxidation.

In the light of these findings, we applied the following temperature variation experiments and defined start‐up procedures that included both oxygen treatment and UV‐irradiation before the addition of MeOH to start photocatalytic MeOH dehydrogenation. In detail, the two oxidative activation protocols were applied as defined in the chapter “Experimental procedure” in the Supporting Information. Depending on the specific purpose of the experiment, one of the two activation conditions (“standard” or “harsh”) was chosen. In this way, we were able to ensure well‐defined and comparable starting conditions for the following experiments.

### 2.3 Effect of Reaction Temperature

**FIGURE 3:**
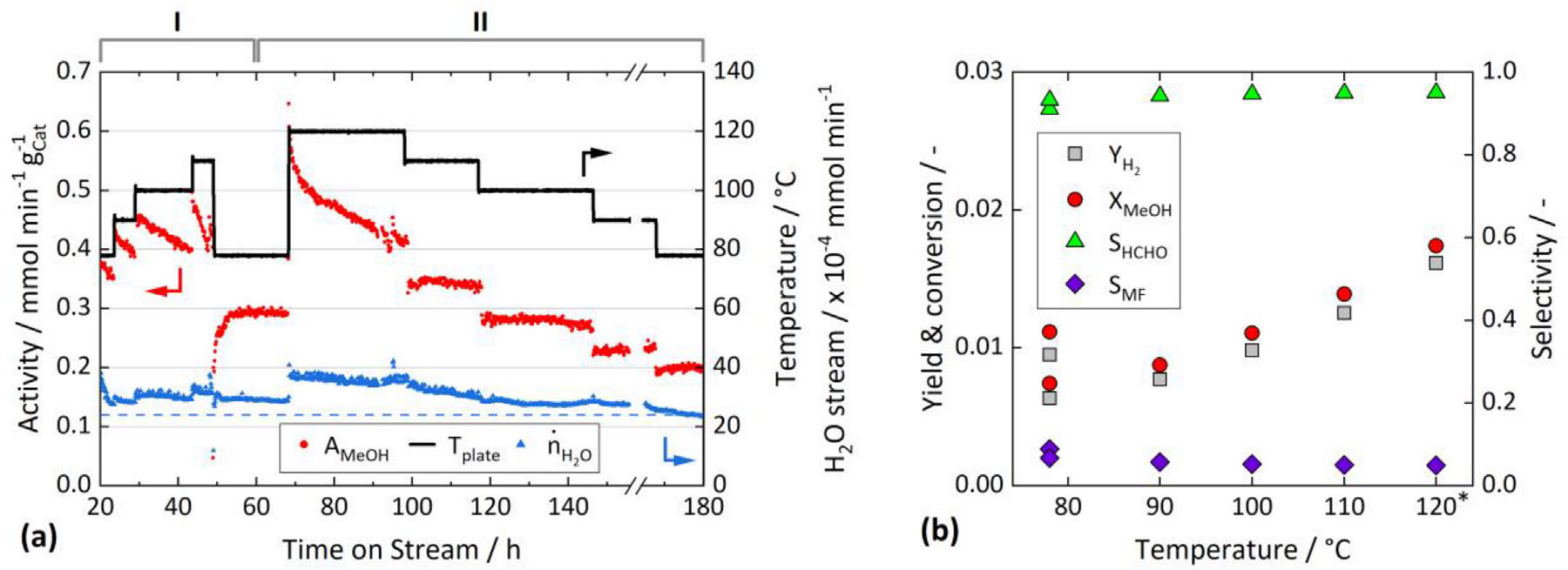
(a) Continuous flow experiment with stepwise increasing temperature from 78°C to 120°C (I) and stepwise decreasing temperature from 120°C to 78°C (II) (dashed line = water stream attributed to methanol feed). (b) Mean values of MeOH conversion, H_2_ yield, and product selectivity for the temperature variation in section II (*non‐stationary operation point at 120°C). Reaction conditions: ‘standard’ activation; m_Cat_ = 0.1006 g, ω = 0.74 mg cm^−2^, E = 255.8 mW cm^−2^, τ = 11.8 s; methanol refilling into the feed tank at 49 h time‐on‐stream.

The reaction temperature is an important parameter as it influences both catalyst activity and deactivation behavior. Figure 3a shows the effect of a stepwise increase of the reaction temperature from 78°C to 110°C (see Figure 3a, section I).

When increasing the temperature gradually in steps of 10 K, catalyst deactivation was observed over time at each new temperature level. The average activity of the respective operation points only marginally increased with temperature. After a prolonged holding time at 78°C with steady but lower activity compared to the initial operation at 78°C, our follow‐up temperature variation started at the highest temperature of 120°C, followed by a stepwise decreasing temperature ramp. Interestingly, only the initially applied highest temperature of 120°C exhibited a visible deactivation (see Figure 3a, section II). For all subsequent lower temperature levels, stable operation points were reached, in contrast to our observations during the upwards temperature ramp (compare Figure 3, sections I and II). Note that the average activity at a given temperature is always lower in section II compared to section I. This suggests that a change in available surface oxygen species (O_sb_) could be the reason for the decreasing activity. We hypothesize that the highest reaction temperature of 120°C results in the highest reduction of O_sb_, which negatively impacts the activity of all following temperature steps. Consequently, after operation at high reaction temperatures like 120°C, the amount of O_sb_ reaches a certain value, with no further reduction when operated at lower temperatures afterwards. In contrast, with increasing temperature, the corresponding state of O_sb_ on the catalyst has to be established over time for every temperature, resulting in the decreasing activity trends shown in Figure 3a, section I. A possible reaction path for the reduction of O_sb_ is the thermal recombination of two surface hydroxyls to water (Equation 12), leaving behind an oxygen vacancy [46]. The increased water quantities detected in the product gas at higher temperatures (see Figure 3a, sections I and II) support this assumption. As described earlier, the accumulation of carbonaceous deposits or CO on the catalyst surface is an additional cause for catalyst deactivation.

Figure 3b shows the mean values for the resulting MeOH conversion, product selectivities and hydrogen yields for the temperature variation experiment in Figure 3a, section II, where stable system operation was reached for all temperatures except for 120°C. Additional reaction data (average productivity, activity and apparent quantum efficiency) are found in Figure S8. For the non‐stationary operation at 120°C (marked ‘*’ in Figure 3b), data from the time interval between 95 h and 98 h were averaged. The two data points at 78°C mark the reference values at the start and end of the experiment in Figure 3a, section II. They show a certain catalyst deactivation over the more than 100 h time‐on‐stream in section II of our temperature variation experiment and reflect the readjustment of Osb after reaction at 120°C.

Analysis of the product distribution in Figure 3b reveals the very remarkable finding that our thermally assisted, photocatalytic MeOH dehydrogenation shows excellent selectivity to formaldehyde and the expected ratio of HCHO and hydrogen formation across the entire temperature range studied. In more detail, a modest exponential increase of MeOH conversion and hydrogen yield is observed with raising temperature. The HCHO selectivity increases from 91.1% at 78°C to 95.0% at 120°C while the methyl formate selectivity decreased from 8.9% to 5.0%. Walenta et al. showed in UHV experiments at temperatures between 235 K and 273 K that the formation of methyl formate (MF) strongly depends on the residence time of formaldehyde on the catalyst surface [24]. As the thermal desorption rate of formaldehyde increases with temperature, the residence time on the catalyst surface decreases, which explains the observed reduced methyl formate formation at higher temperatures. Furthermore, the diffusion of surface‐bound hydrogen to the Pt‐clusters increases with higher temperatures, resulting in more available O_sb_ for methoxy and formaldehyde formation (see Equations 3 and 4). This effect is expected to accelerate the overall reaction. The photon stream was kept constant throughout the temperature variations. As a result, the total AQE follows the described trends of the conversions and product yields (see Figure S8 for details).

### 2.4 Variation of Irradiance and Residence Time

**FIGURE 4:**
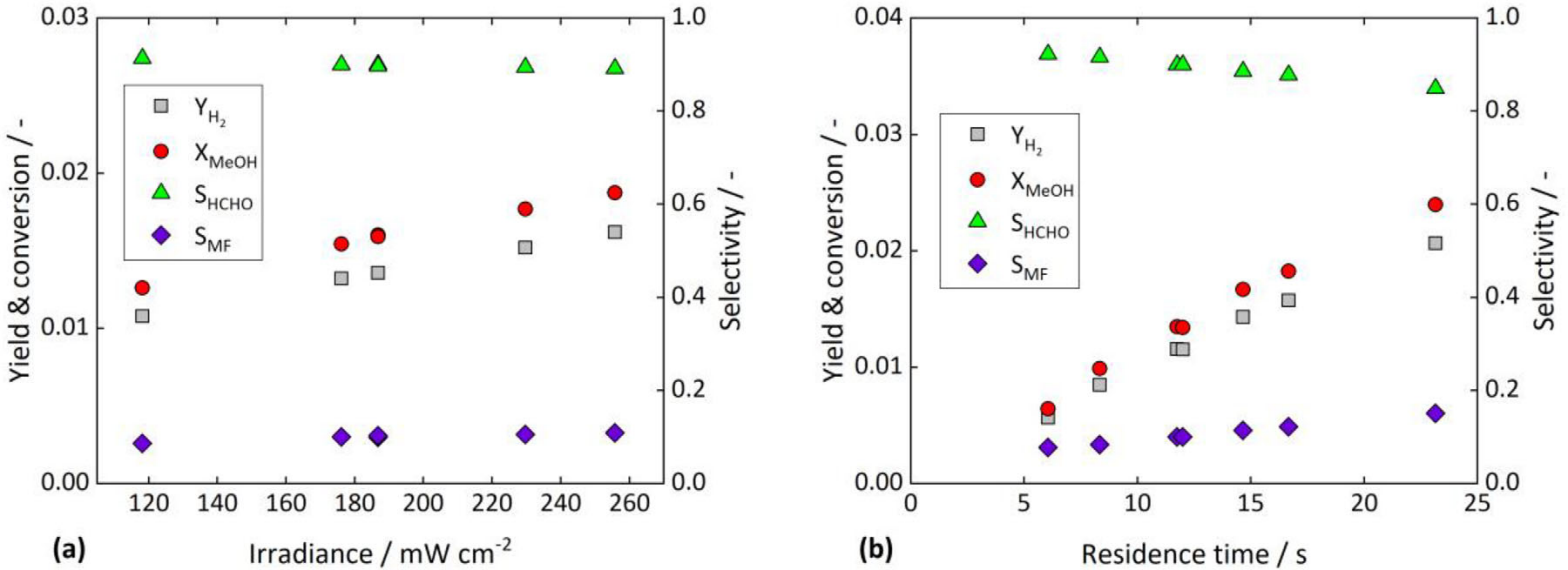
Variation of irradiance (a) and residence time (b) and resulting MeOH conversion, H_2_ yield and product selectivity (*non‐stationary operation point). The data were treated as shown in Figure S7. Reaction conditions: ‘standard’ activation conditions; (a): m_Cat_ = 0.1241 g, ω = 0.91 mg cm^−2^, τ = 11.8 s, T_Cat_ = 78°C; (b): m_Cat_ = 0.1006 g, ω = 0.74 mg cm^−2^, E = 207.8 mW cm^−2^, T_Cat_ = 78°C.

In the next set of experiments, we studied the influence of irradiance on the conversion, product selectivity, and hydrogen yield in continuous photocatalytic MeOH dehydrogenation (Figure 4a). Additional reaction data (productivity, activity, and apparent quantum efficiency) can be found in Figure S9, for all irradiation intensities. To compare data from different time‐on‐stream despite the mild deactivation observed during the temperature variation (see above), the data of the following experiments were processed to compensate for this slight activity loss according to a procedure explained in Figure S7.

Our results show that higher irradiance leads to an increase in activity and consequently MeOH conversion. The latter increases linearly from 1.3% to 1.9% with increasing irradiation from 120 to 255 mW cm^−2^. Remarkably, the observed MeOH conversion of 1.9% is 60 times higher than the thermodynamic equilibrium conversion for the MeOH dehydrogenation reaction at 78°C for a corresponding, purely thermally activated reaction (see Figure S1 for details). The HCHO selectivity was influenced only to a negligible degree and remained in the range of 89.1% to 91.4%. A maximum HCHO yield of 1.7% was reached at the highest irradiance of 255 mW cm^−2^. The total and HCHO‐related AQE decreases with higher irradiance, while the MF‐related AQE stays fairly constant at around 0.9% (see Figure S9 for details).

In the next set of experiments, the residence time in the photocatalytic reactor was varied. The resulting conversions, product selectivities and hydrogen yields are shown in Figure 4b. In addition, Figure S10 provides the AQE and productivity data. With increasing residence time, MeOH conversion increases linearly. Doubling the residence time resulted in an almost doubled MeOH conversion in the investigated range. With higher methanol conversion, the selectivity to MF increases slightly because of the higher amount of formaldehyde and methoxy species on the surface. The highest HCHO yield of 2.0% is found for the longest tested residence time of 23.3 s. Total AQE drops with increasing residence time because MF formation consumes one additional incoming photon (see Equations 8 and 9) (see Figure S10 for details).

### 2.5 Influence of Oxygen Co‐Dosing

**FIGURE 5:**
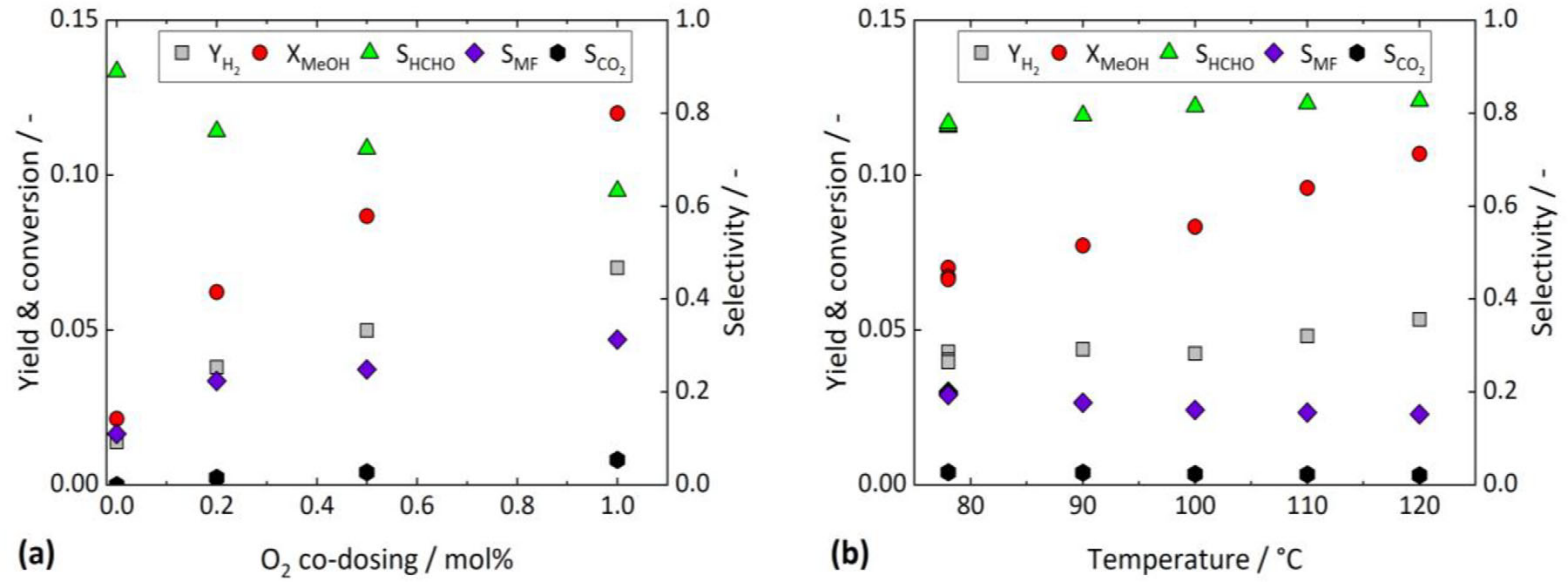
MeOH conversion, H_2_ yield and product selectivities at varying oxygen concentrations (a) and temperatures (b). Activation procedure: ‘harsh’ activation conditions, thermally reduced catalyst. Reaction conditions: E = 255.8 mW cm^−2^, τ = 11.8 s, c_MeOH_ = 10 mol%; (a): T_Cat_ = 78°C, m_Cat_ = 0.1521 g, ω = 1.12 mg cm^−2^; (b): c_O2_ = 0.5 mol%, m_Cat_ = 0.1220 g, ω = 0.90 mg cm^−2^.

As shown by the activation experiments, oxygen has a strong influence on the overall activity of the photocatalytic system. Such positive effects of co‐adsorbed O_2_ on the activity of a TiO_2_ photocatalyst have also been described in surface science experiments in the literature [29]. Therefore, a continuous co‐dosing of small amounts of oxygen (≤1 mol%) was implemented into our photocatalytic MeOH dehydrogenation experiment. Our intention was to continuously suppress the above‐described deactivating effects (loss of O_sb_, carbonaceous deposits, CO poisoning) while still avoiding methanol oxidation (forming water instead of hydrogen as coupling product) as much as possible. To introduce as much surface oxygen as possible from the beginning of the experiment, we used the ‘harsh’ activation procedure (as described in the experimental procedure, Supporting Information for the following experiments. The resulting MeOH conversions, product selectivities and H_2_ yields are shown in Figure 5a.

Most interestingly, the MeOH conversion increased with increasing oxygen concentration in the reaction mixture from 2.1% (no O_2_ co‐dosing) to 12.0% (1.0 mol% O_2_ co‐dosing). Even an addition of just 0.2 mol% O_2_ led already to a threefold increase in MeOH conversion compared with the oxygen‐free conditions. The overall hydrogen yield is increasing by several factors as well, which clearly shows that hydrogen oxidation to water is not the main reaction pathway. Details on the formation of water are shown in Figure S13a. In brief, for the operation without added oxygen, only trace amounts of water are detected, which are likely to originate from the methanol feed. With O_2_ co‐dosing of 0.2 mol%, a H_2_ to H_2_O ratio of 1.56 was found, which changed to 1.14 for an O_2_ co‐dosing of 1 mol% O_2_.

By comparing our experimental data under dark and irradiated conditions (details see in Figure S14), we were able to quantify the purely thermal contribution to MeOH oxidation on Pt/TiO_2_ under the applied reaction conditions with oxygen co‐dosing. The main product of the dark condition experiment was found to be MF, which is likely formed as a thermal coupling product from HCHO via a Tischenko‐type reaction (Equation 10). As seen from Figure 5a, total oxidation of MeOH is observed only to a very small extent, e.g., at O_2_ co‐dosing of 1 mol% the CO_2_ selectivity stays below 5.4%. Instead, the selectivity to MF increases with higher oxygen concentration from 11.0% at oxygen‐free conditions to 31.3% at 1 mol% O_2_ co‐dosing. Consequently, the HCHO selectivity goes down from 89.0% (no O_2_ co‐dosing) to 63.3% (1 mol% O_2_ co‐dosing). We hypothesize that oxygen co‐dosing results in an increased O_sb_ concentration, which in turn leads to a higher surface coverage with methoxy and adsorbed formaldehyde species, enhancing MF formation (see Equations 8 – 10) [42]. Doubling the residence time to 23.5 s while co‐dosing 1 mol% oxygen led in our experiments to a peak methanol conversion of 17.8% combined with a hydrogen yield of 11.2% and a further increase in MF yield as detailed in Figure S15. Although [62, 63, 64] HCHO and hydrogen are the target products of our photocatalytic methanol dehydrogenation process, the simultaneous formation of MF is also of great interest, as MF has high technical value and its formation thus represents another striking feature of this thermally assisted photocatalytic methanol dehydrogenation process.

The influence of reaction temperature at a fixed oxygen co‐dosing of 0.5 mol% is depicted in Figure 5b (see Figure S13b for details). As expected from our previous temperature variation experiments, increasing reaction temperatures resulted in an increasing MeOH conversion, here from 6.8% to 10.7% by going from 78°C to 120°C reaction temperature. The selectivity for HCHO and MF formation is also in line with our observations for the oxygen‐free system (comparison with Figure 3b): Increasing temperature reduces the relative MF formation due to faster thermal desorption of formaldehyde from the catalyst surface. In contrast to the oxygen‐free experiment, the H_2_ yield increases only marginally with rising temperatures. We hypothesize that higher temperatures favor the MeOH oxidation route instead of the direct dehydrogenation, which is in good agreement with the increasing water formation observed (for details see Figure S13b). Furthermore, O_2_ co‐dosing also shows a beneficial effect on catalyst stability, in particular in experiments with pronounced temperature ramping (see Figure S16 for details). This observation supports our hypothesis that a reduction of O_sb_ is one relevant reason for catalyst deactivation in the oxygen‐free operation.

### 2.6 Long‐Term Stability Without and With Oxygen Co‐Dosing

**FIGURE 6:**
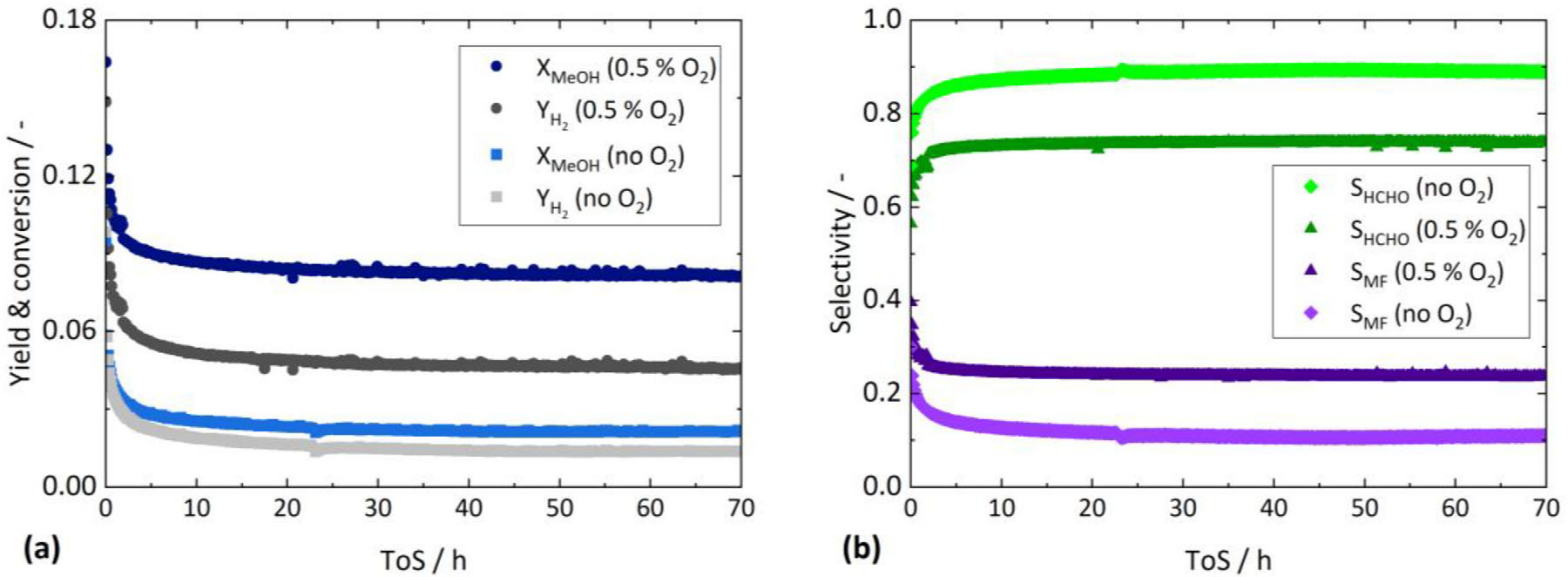
MeOH conversion and H_2_ yield (a) as well as product selectivities (b) during long‐term stationary operation (’harsh’ activation conditions; m_Cat_ = 0.1521 g, ω = 1.12 mg cm^−2^, thermally reduced, E = 255.8 mW cm^−2^, T_Cat_ = 78°C, τ = 11.8 s).

These results encouraged us to investigate our thermally assisted photocatalytic MeOH dehydrogenation also in continuous long‐term experiments under steady‐state conditions. Figure 6 shows MeOH conversion and H_2_ yield data for a run over 70 h time‐on‐stream at 78°C with an irradiation of 255.8 mW cm^−2^. We compare two runs that only differ from the fact that one (circles and triangle symbols in Figure 6a,b, respectively) uses an O_2_ co‐dosing of 0.5 mol%, while the other (boxes and diamonds) is operated without O_2_ co‐dosing.

After a run‐in period of approx. 20 h, stationary operation is reached for both experiments with no significant catalyst deactivation over the following 50 h time on stream (Figure 6a). The average MeOH conversion in this phase is 2.2% without and 8.3% with 0.5 mol% oxygen co‐dosing. The selectivities to HCHO (73.7% with and 89.1% without co‐dosing) and MF (23.7% with and 10.9% without co‐dosing) are also stable after the run‐in period (see Figure 6b) and follow the trends described above. The hydrogen yield in oxygen‐free conditions is 1.4%. It increases to 4.7% for the steady operation point in the case of O_2_ co‐dosing. The higher discrepancy between MeOH conversion and H_2_ yield under co‐dosing operation is attributed to some partial oxidation of MeOH to HCHO instead of MeOH dehydrogenation, as discussed above. Overall, both long‐term experiments demonstrate an excellent stability of the coated Pt/TiO_2_ photocatalyst in continuous operation with and without oxygen co‐dosing, which is critical for a further scale‑up of the here‐proposed MeOH dehydrogenation technology.

## 3 Conclusion

This study represents—to the best of our knowledge—the first successful attempt of performing the thermally‐assisted photocatalytic dehydrogenation of methanol in a continuous reactor over an extended operation period (70 h time‐on‐stream under steady‐state conditions). Using a spray‐coated Pt on TiO_2_ catalyst, very mild reaction conditions (<120°C, atmospheric pressure) and—in some of our experiments—oxygen co‐dosing for drastically enhancing the catalyst activity, our work establishes a new, robust and highly selective method for continuous MeOH conversion into formaldehyde, methyl formate and hydrogen with conversion levels that far exceed the thermodynamic equilibrium of the respective purely thermally activated processes. Compared to the limited literature on continuous photocatalytic methanol dehydrogenation, the achieved methanol conversions and formaldehyde yields are favorable. While higher conversions are reported for batch systems, continuous‐flow studies remain scarce and often lack comparable process‐level metrics, underlining the relevance of the present benchmark study. From our results, the following influences of key operation parameters on the thermally assisted photocatalytic MeOH dehydrogenation can be deduced:

1. To achieve maximum MeOH conversion under oxygen‐free conditions, high reaction temperature, residence time, and irradiance should be combined.
2. Residence time and reaction temperature are the most influential parameters to impact reaction selectivity, with high residence time and low temperature shifting selectivity to MF instead of HCHO.
3. To maximize apparent quantum efficiency (AQE), a combination of low irradiance, residence time, and high temperature is favorable.
4. Oxygen co‐dosing (up to 1 mol%) is a promising strategy to strongly increase MeOH conversion (by a factor of up to 5) and product yields without significantly complicating the process design of the thermally assisted photocatalysis; methanol dehydrogenation remains the dominant reaction pathway, while hydrogen oxidation to water is largely suppressed, accompanied by a selectivity shift toward methyl formate formation.

We anticipate significant technical interest in the MeOH valorization technology presented here. Future investigations should focus on optimizing the photocatalyst material and the reaction conditions, e.g., to further reduce oxidative methanol conversion under operating conditions that use oxygen co‐dosing for surface activation.

## Conflicts of Interest

The authors declare no conflict of interest.

## Supporting information




**Supporting File**: advs76842‐sup‐0001‐SuppMat.docx.

## Data Availability

The data that support the findings of this study are openly available in Zenodo at https://zenodo.org/, reference number 10.5281/zenodo.18851601.
