# Supplementary material for: Thermally Assisted Photocatalytic Dehydrogenation of Methanol in Continuous Steady‐State Operation
Source: Adv Sci (Weinh). 2026 Jul 27:e76842. Online ahead of print. doi: 10.1002/advs.76842 (PMC13403887; doi:10.1002/advs.76842)
Supplement: Supplementary file 1 — Supporting File: advs76842‐sup‐0001‐SuppMat.docx. [file ADVS-9999-e76842-s001.docx]

**Supporting Information**

**Thermally assisted Photocatalytic Dehydrogenation of Methanol in Continuous Steady-state Operation**

Stubenrauch, Florian; ^[b]^ Nathrath, Phillip; ^[c]^ Schörner, Markus; ^[b]^ Mahayni, Yazan; ^[b]^ Fritsch, Birk; ^[b]^ Hutzler, Andreas; ^[b]^ Wasserscheid, Peter; ^[a,b,c]^ Bösmann, Andreas;^[c]^ Schühle, Patrick^[a,c]^*

[a] P. Wasserscheid, P. Schühle
Forschungszentrum Jülich GmbH
Institute for a sustainable Hydrogen Economy (IHE),
Wilhelm-Johnen-Straße, DE-52428 Jülich, Germany
* p.schuehle@fz-juelich.de

[b] F. Stubenrauch, M. Schörner, Y. Mahayni, B. Fritsch, A. Hutzler, P. Wasserscheid
Forschungszentrum Jülich GmbH
Helmholtz-Institut Erlangen-Nürnberg for renewable Energy (IEK‑11)
Cauerstr. 1, DE-91058 Erlangen, Germany

[c] P. Nathrath, P. Wasserscheid, A. Bösmann, P. Schühle

Friedrich-Alexander-Universität Erlangen-Nürnberg

Institute of Chemical Reaction Engineering

Egerlandstr. 3, DE-91058 Erlangen, Germany

## S0 Methods

**Materials:** Chloroplatinic acid (H_2_PtCl_6_, Sigma Aldrich), polyvinylpyrrolidone (PVP, MW 40 000, Sigma Aldrich), and sodium borohydride solution (NaBH_4_, 33 mM in 10 mM NaOH, Acros Organics) were used as received. MeOH (Emplura & Macron fine chemicals, purity ≥99.5 %) and ethanol (TechniSolv, purity ≥99.5 %) were bought from Merck. The applied photocatalyst was based on the titanium (IV) oxide (TiO_2_) Aeroxide® P25 (Thermo Scientific, lot: A0407014 & A0455375).

**Catalyst preparation:** To produce the applied Pt/TiO_2_ catalyst powder, a chemical reduction procedure was adapted from previous work by some of us [56]. At first, the capping agent PVP was dissolved in 50 mL of Millipore water. This solution was ultrasonicated for 15 min to ensure the homogenization of the solution. An amount of 52 mg of H_2_PtCl_6_ was added to the PVP solution followed by ultra‑sonication for 1 min. The obtained solution was magnetically stirred at 1500 rpm and the reducing agent was rapidly injected. The NaBH_4_ solution for reduction was prepared separately in 10 mM NaOH to limit decomposition of NaBH_4_ by hydrolysis. To yield Pt nanoparticles with ~2 nm particle size, a volume of 1.1 mL of the reducing agent was added. After 1 min of stirring, the nanoparticles were immobilized on the P25 support and left overnight under moderate stirring to ensure the homogeneous distribution of the nanoparticles on the support. A loading of 0.4 wt.% Pt was targeted for all catalyst preparations by the addition of the appropriate amount of P25 support (typically 5 g). Finally, the solvent was removed in a rotary evaporator (1^st^ step: 250 mbar / 80 °C; 2^nd^ step: 35 mbar / 80 °C) to obtain the catalyst for characterization and catalytic testing. The obtained catalyst powder was ground and then used without further purification. For comparison, a thermally reduced Pt/TiO₂ catalyst powder with a similar Pt loading was prepared with the same Pt precursor and TiO₂ support (see ESI, Section S8, for details).

**Catalyst immobilization:** The powder catalyst was immobilized on planar stainless-steel substrates (1.4571, 170 x 80 x 1,5 mm) to make it applicable for the photocatalytic flow reactor. Prior to the coating step, the steel plates were roughened with 120 grid sandpaper for better adhesion. For catalyst powder deposition, a spray-coating process was used resulting in 136 cm^2^ of coated active area. For this coating step, an amount of catalyst material proportional to the desired mass loading was weighed into a beaker and 30 mL of ethanol was added. This slurry was then ultrasonicated for 5 min to achieve good dispersion. Afterwards, the suspension was manually sprayed onto the substrate plates in several layers using a hand-held spray gun. In between each layer and after the final layer, the coated plate was dried at 100 °C for 5 min on a hot plate. After cooling, the catalyst plate was used without further treatment in the flow reactor.

**Photocatalytic flow reactor setup:** The photocatalytic experiments were conducted in a continuously operating photocatalytic laboratory unit that has been described in detail elsewhere [57]. An overview of the applied experimental configuration and a visual representation of the flow reactor can be found in Figure S2 of the ESI. The rectangular flow reactor has a quartz glass window for UV irradiation of the equipped catalyst-coated plate. The flow reactor was heated from the bottom side using electrical heating. Temperature control was realized using a thermocouple attached to the catalyst-coated plate. For UV-irradiance, an LED array from Neumüller Elektronik GmbH was used. The latter was equipped with 56 UV-A LEDs (CUN66A1B Seoul Viosys) with a peak wavelength of λ_peak_ = 365 nm and an optical output of 1.1 W per single LED. In a previous study we showed the radiometry data on the irradiation intensity and distribution throughout our reactor [57]. Vaporized MeOH is fed into the reactor by a Bronkhorst CEM evaporator system with N_2_ as carrier gas. The MeOH stream was controlled by a Coriolis flow meter. All gas streams (N_2_, O_2_ and H_2_) were controlled by mass flow controllers (Bronkhorst El-Flow Prestige).

**Experimental procedure:** To perform a continuous photocatalytic run, the freshly prepared catalyst plate was inserted into the reactor. The reactor was fully assembled and purged with N_2_ for 5 min at 500 mL_N_ min^-1^. Afterwards, an activation phase *(vide infra)* was conducted using the ‘standard’ or the ‘harsh’ catalyst activation treatment. The standard protocol used an O_2_ concentration of 3 vol.% and an irradiance of 186.8 mW cm^-2^, while the harsh conditions comprised 21 vol.% O_2_ and an irradiance of 255.8 mW cm^-2^. Both treatments were conducted for 3 h at 78 °C.

After this activation procedure, the feeding of 10 vol.% MeOH in N_2_ was started. The temperature of the evaporator was always kept constant at 70 °C. Finally, the photocatalytic reaction was started by turning the LED-array on. In some cases, data were collected without UV-irradiation for one hour before illumination to allow a comparative investigation of the catalyst activity under dark conditions.

**Product analytics:** The reaction products were analyzed by online gas chromatography (Micro-GC Fusion, Inficon). Equipped with three modules, the applied GC allowed for a complete quantitative and qualitative analysis of the entire product gas composition.

**Catalyst characterization:** High-angle annular dark field (HAADF) scanning transmission electron microscopy (STEM) (Thermo Fisher Scientific Talos F200i (S)TEM at 200 kV acceleration voltage) of the applied catalyst was conducted in combination with energy-dispersive X-ray spectroscopy (EDXS) mapping (Dual Bruker XFlash 6 | 100 EDS detectors). For these investigations, the catalyst particles were drop-casted on a holey-carbon on gold TEM grid (Plano). Image analysis was carried out using the software FIJI [58].

**Calculations:** To calculate MeOH conversion, the consumed amount of MeOH was determined from an analytical assessment of the formed products (see Equation 13).

|  | $\dot{n}_{MeOH, consumed} = \dot{n}_{0,MeOH}-\dot{n}_{MeOH} =\Sigma\dot{n}_{j}\cdot N_{C} -\Sigma\dot{n}_{j, 0}\cdot N_{C}$ | (13) |
| --- | --- | --- |

| Conversion of MeOH: | $X_{i}=\frac{\dot{n}_{MeOH,consumed}}{\dot{n}_{0,MeOH}} \left[ \frac{mol}{mol} \right]$ | (14) |
| --- | --- | --- |
| Product selectivity: | $S_{i,j}=\frac{\dot{n}_{j}-\dot{n}_{j,0}}{\dot{n}_{MeOH,consumed}}\cdot\frac{\nu_{MeOH}}{\nu_{j}} \left[ \frac{mol}{mol} \right]$ | (15) |
| Product yield: | $Y_{i,j}=\frac{\dot{n}_{j}-\dot{n}_{j,0}}{\dot{n}_{0,MeOH}}\cdot\frac{\nu_{MeOH}}{\nu_{j}} \left[ \frac{mol}{mol} \right]$ | (16) |

Conversion, product selectivity and product yield were calculated accordingly considering MeOH as the only carbon feedstock molecule and with the index j representing the different products (see Equations 14-16). The selectivities of all products sum up to one (100%) as a mathematical consequence of our calculation procedure.

According to the stoichiometries of our reaction network, the amount of H_2_ produced should equal the sum of the amount of HCHO (including HeFal) and twice the amount of MF:

|  | $\frac{\dot{n}_{HCHO}+ 2 \dot{n}_{MF}}{\dot{n}_{H_{2}}}= 1$ | (17) |
| --- | --- | --- |

Catalyst activity and productivity were calculated in terms of molar feedstock consumption and product formation rate per mass of catalyst, respectively.

| Activity: | $A_{MeOH}=\frac{\dot{n}_{MeOH,consumed}}{m_{cat}} \left[ \frac{mmol}{g_{Cat} min} \right]$ | (18) |
| --- | --- | --- |
| Productivity: | $P_{j}=\frac{\dot{n}_{j}-\dot{n}_{j,0}}{m_{cat}} \left[ \frac{mmol}{g_{Cat} min} \right]$ | (19) |

The apparent quantum efficiency (AQE) was calculated as the fraction of the incoming photon-stream that was consumed in product formation. We assumed a one photon process for HCHO formation (N_hν_ = 1), and a two photon-process for MF formation (N_hν_ = 2) [18,31].

| AQE: | | $AQE=\frac{\dot{n}_{j}-\dot{n}_{j,0}}{\dot{n}_{h\nu}}\cdot N_{h\nu} \left[ \% \right]$ | (20) |
| --- | --- | --- | --- |
|  |  |  |  |

**Statistical Analysis:**

The shape of the Pt particles in HAADF-STEM analysis appears to be roughly spherical or elliptical. From different images a total of 327 particles were counted for size analysis. From the areas measured the diameter of volume equivalent spheres was calculated. The resulting histogram with Gaussian fitted distribution (R²adj. = 0.94, fitting equation, parameters and respective errors in Table S1) can be seen in Figure 1c.

Table S1: Gaussian fit to Pt-particle size distribution from TEM evaluation.

| **Parameter** | **Value** | **Error** |
| --- | --- | --- |
| **Fitted equation** | $y_{0} +\frac{A}{w \sqrt{\frac{\pi}{2}}}\exp\left( -2\frac{\left( x-x_{c} \right)^{2}}{w^{2}} \right)$ |  |
| **y_0_** | 0.3867 | 0.4394 |
| **x_c_** | 2.0632 | 0.0162 |
| **w** | 0.7778 | 0.0370 |
| **A** | 25.5934 | 1.2881 |
| **FWHM** | 0.9158 | 0.0436 |
| **R²_adj._** | 0.9399 |  |

Each operation point in the dehydrogenation studies was determined from one experiment under the respective conditions. Analysis of reaction product composition was performed via online GC on a stationary operation point by using a sample size of three, meaning that three product samples were measured repetitively in the GC. Mean values were calculated and are given in the figures.

## S1 Thermodynamic equilibrium data

The equilibrium data shown in Figure S1 was calculated with Aspen Plus V12.1 (method RK-SOAVE). In the temperature range of photoreactor operation only very low MeOH conversion (<0.2 %) is achievable through pure thermal reaction pathways even if the system is diluted with 90 mol% N_2_.


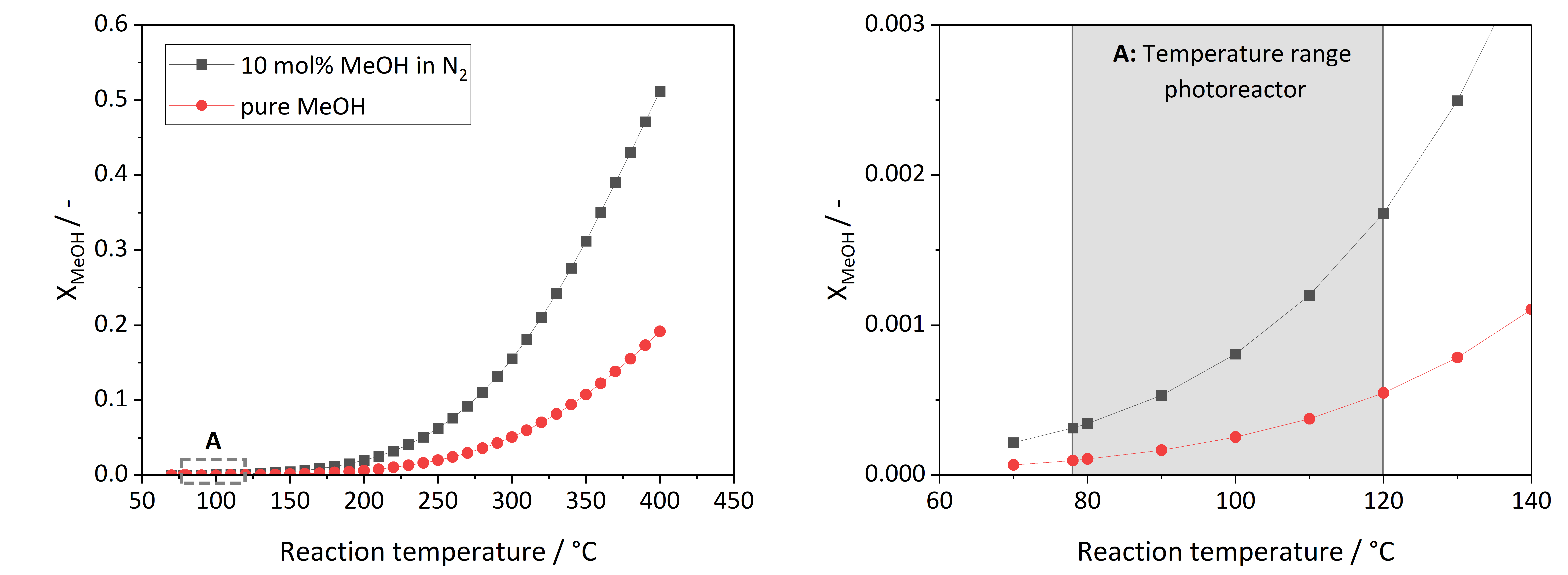


Figure S1: Simulated equilibrium data for the water-free methanol dehydrogenation to formaldehyde and H_2_.

## S2 Overview photoreactor plant


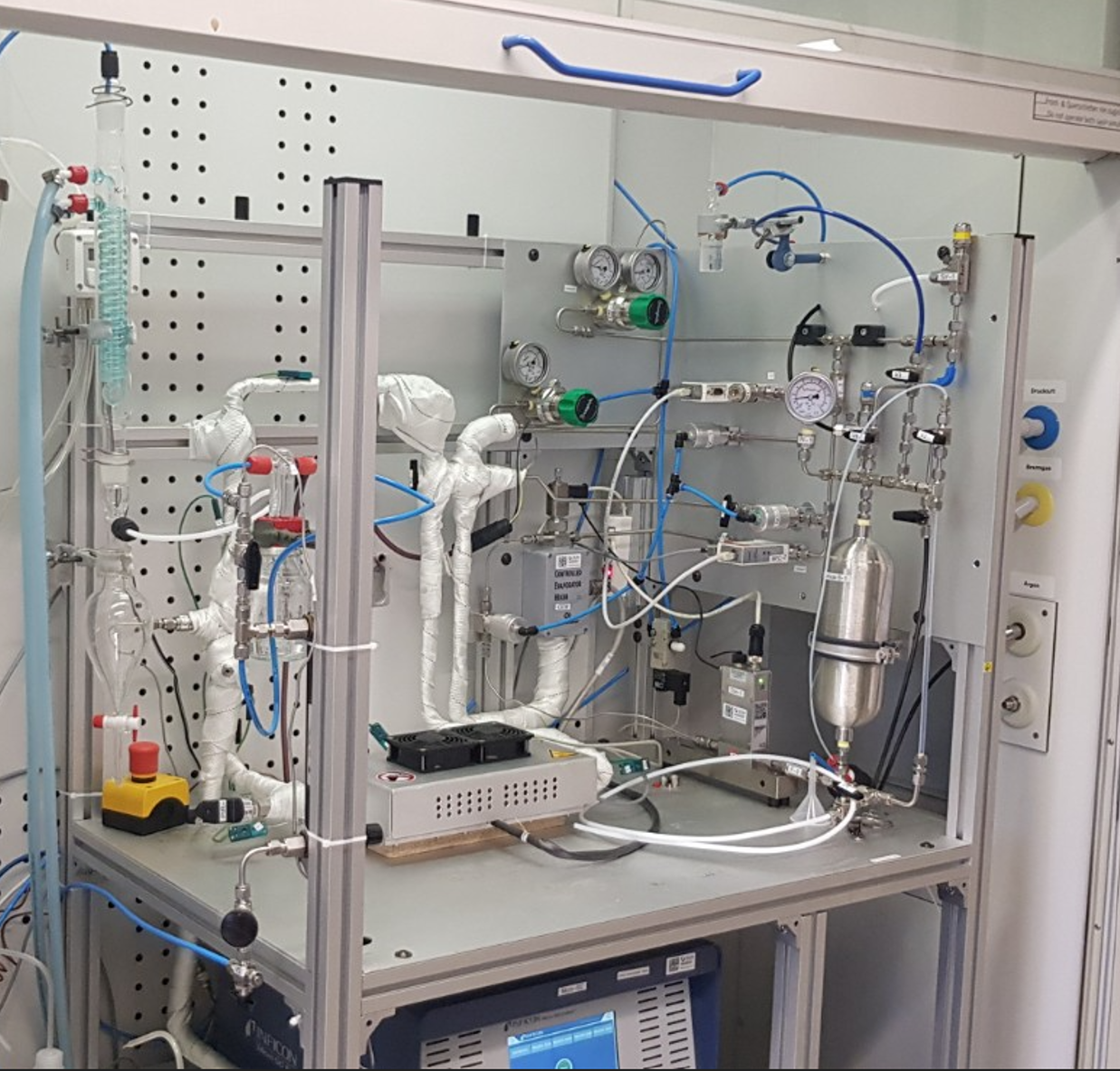

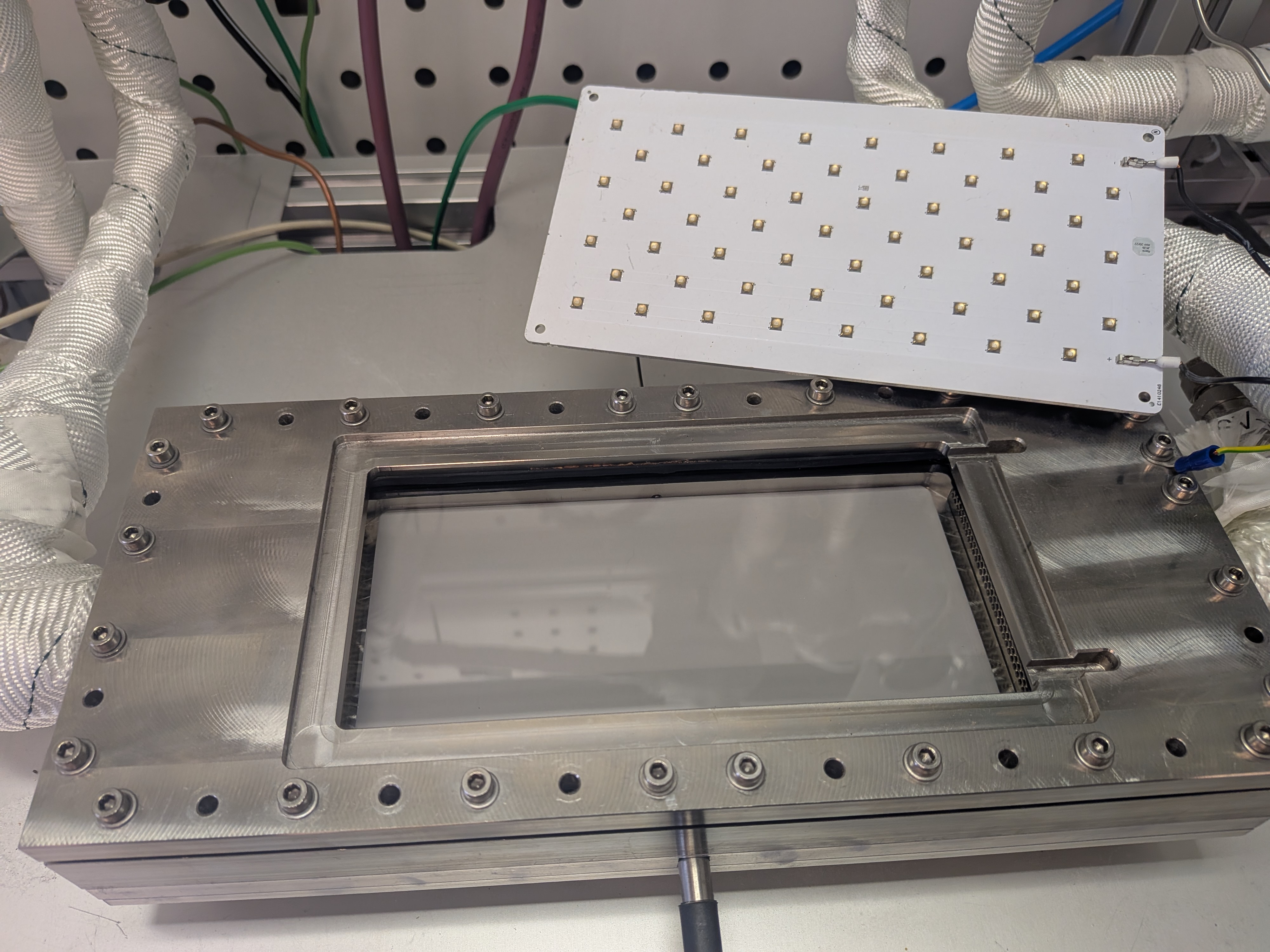


**1**

**2**

**3**

**4**

**5**

**a**

**b**

**c**

**5**

**6**

**7**

Figure S2: Overview of the laboratory plant and photoreactor: 1) gas supply; 2) feed tank; 3) Coriolis mass flow meter; 4) evaporator; 5) flow reactor with a) UV-LED array, b) glass window, c) coated plate; 6) micro-GC; 7) product condenser.

## S3 Catalyst Activation

**Experiment with alternative treatment conditions:**

Figure S3: Catalyst activities after treatments under different conditions: light blue: 3 vol.% H_2_ in N_2_ at 100 °C, purple: 3 vol.% H_2_ in N_2_ at 100 °C with irradiation (186.8 mW cm^‑2^); orange: 3 vol.% O_2_ + 6 vol.% MeOH in N_2_ with irradiation (186.8 mW cm^‑2^) at 78 °C. Reaction conditions for activity test: 10 vol.% MeOH in N_2_, E = 186.8 mW cm^‑2^, T = 78°C, τ = 11.8 s.

**Repeated oxidative catalyst (re-)activation:**

Figure S4 shows the activity of the same catalyst and equal reaction conditions after initial oxidative pretreatment (1^st^ Reactivation) and four additional oxidative pretreatment cycles (2^nd^ – 5^th^ Reactivation). The duration of the oxidative activation was 1 h for the 1^st^ reactivation, 3 h for the 2^nd^, 3^rd^ and 5^th^ reactivation and >12 h for the 4^th^ reactivation. For the three cycles with the same activation duration, only neglectable differences in steady state activity are observed. Overall, longer oxidative activation leads to slightly higher activities after the initial run-in period.

Figure S4: Activity data from multiple experiments of identical conditions and catalyst. Reactivation method ‘standard’ with varying duration of 1 h in 1^st^, 3 h in 2^nd^, 3^rd^ & 5^th^, and >12 h in 4^th^ reactivation period; m_Cat_ = 0.1241 g; ω = 0.9125 mg cm^‑2^; τ = 11.8 s; T = 78 °C; E = 186.8 mW cm^‑2^.

**Ratio of H_2_O to CO_2_ during the oxidative activation treatments:**

Figure S5: Ratio of H_2_O to CO_2_ during the two activation phases. Activation conditions: 3 vol.% O_2_ in N_2_ at T = 78 °C and E = 186.8 mW cm^‑2^. Reaction conditions for activity test: 10 vol.% MeOH in N_2_, E = 186.8 mW cm^‑2^, T = 78 °C, τ = 11.8 s.

## S4 Thermogravimetric analysis of Pt/TiO_2_ catalyst

The measurements were carried out with a TA Instruments SDT 2960 coupled with a Pfeiffer Vacuum Thermostar (GSD 300 T1) mass spectrometer. TGA measurements were conducted by heating the sample to 800 °C (5 K min^-1^) in synthetic air and the weight of the sample as well as the gas phase composition was continuously monitored.

**(a)**

**(b)**

Figure S6: TGA-MS measurements of the fresh Pt/TiO_2_ catalyst (a) and spent Pt/TiO_2_ catalyst after approx. 1064 h total time on stream (b).

Figure S6 shows the TGA-MS results of a freshly prepared and a spent Pt/TiO_2_ catalyst. Both samples exhibit some mass loss in the temperature range 50-300 °C attributed to desorption of water. In the fresh catalyst no CO_2_ formation was measured, whereas the spent catalyst showed significant CO_2_ formation in the temperature range 180-400 °C. The peak center of this CO_2_ formation is around 287 °C, which could indicate adsorbed formate species or polymeric deposits that are thermally decomposed to CO_2_. The overall mass loss of the spent catalyst is quantified as 4.8 %, more than double of the freshly prepared catalyst (2.3 %).

## S5 Parameter influences - deactivation compensation

To compensate the deactivation during the parameter variation experiments (for irradiance and residence time variation under oxygen free conditions) a method was applied, that subtracts the effect from the data by fitting the decay. During each parameter experiment, a referenceable operation point was conducted before and after the variations. A power function fit is then applied to these reference points. The very last data points are taken as an approximation to stationary operation. By subtraction of the exponential part, a set of data with activity loss compensation is achieved. The method is graphically explained below.

Figure S7: Graphical summary of data evaluation to compensate catalyst deactivation in parameter variation experiments.

## S6 Parameter variation - additional data

1. Temperature (no compensation of deactivation behavior was conduted according to paragraph S6 due to reasons discussed):

Figure S8: Additional reaction data from the temperature variation experiment. ‘standard’ activation conditions; m_Cat_ = 0.1006 g, ω = 0.74 mg cm^-2^, E = 255.83 mW cm^-2^, τ = 11.8 s.

1. Irradiance influence (compensated according to paragraph S6):

Figure S9: Additional reaction data for different irradiation intensities. ‘standard’ activation conditions; m_Cat_ = 0.1241 g, ω = 0.91 mg cm^-2^, τ = 11.8 s; T_Cat_ = 78 °C.

1. Residence time variation (compensated according to paragraph S6):

Figure S10: Additional reaction data for different residence times. ‘standard’ activation conditions; m_Cat_ = 0.1006 g, ω = 0.74 mg cm^-2^, E = 255.83 mW cm^-2^, T_Cat_ = 78 °C.

## S7 Comparison of chemically and thermally reduced Pt/TiO_2_ catalyst

We compared our chemically reduced Pt/TiO_2_ to thermally reduced Pt/TiO_2_ catalyst. The BET surface area of the P25 TiO_2_ support material was determined as 53 m^2^ g^-1^ via N_2_-physisorption. The latter was synthesized via wet impregnation with H_2_PtCl_6_, calcination and subsequent reduction at 440 °C for 2 h in 10 vol.% H_2_/N_2_ stream. Both catalysts exhibit similar Pt-loading of approx. 0.4 wt.% as verified by ICP-AES. The exact ICP values along characteristics of the coated plates are displayed in Table S2.

| **Catalyst** | **Pt loading / wt%** | **Catalyst mass / g** | **Pt mass / mg** |
| --- | --- | --- | --- |
| Thermally reduced (TR) | 0.40 | 0.1220 | 0.488 |
| Chemically reduced (CR) | 0.39 | 0.1241 | 0.484 |

Table S2: Overview of thermally reduced (TR) and chemically reduced (CR) catalyst coated plates used in the photoreactor (Pt loading determined via ICP-AES).

Figure S11 shows the results in terms of MeOH conversion, H_2_ yield and product selectivities for an experiment at reference conditions with both catalysts. The selectivities to HCHO and MF are in a similar range for both systems, with a slightly higher affinity towards MF formation for the TR system. This can be attributed to the higher MeOH activity of the TR catalyst compared to the CR system. The H_2_ productivity is around 0.35 mmol g_Cat_^-1^ min^-1^ for the CR system and around 0.37 mmol g_Cat_^-1^ min^-1^ for the TR system. Due to the very similar performance but simpler preparation method, we conducted all experiments with oxygen co-dosing using the thermally reduced catalysts.


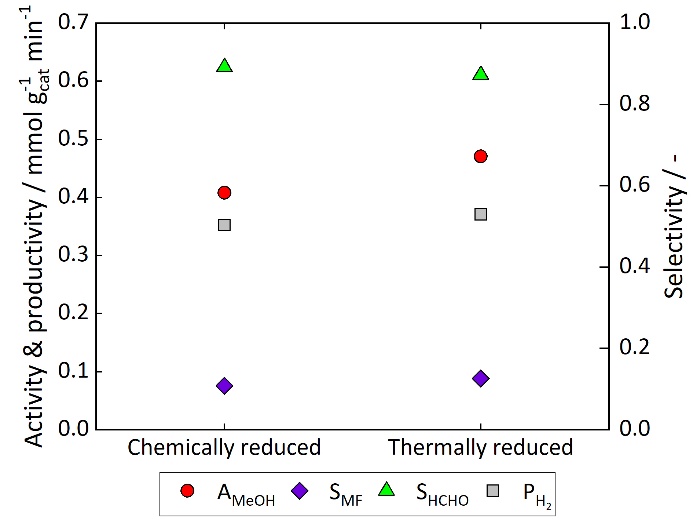


Figure S11: MeOH conversion, H_2_ yield and product selectivity of thermally and chemically reduced Pt/TiO_2_ catalyst. Reaction conditions: catalyst data see Table S2, τ = 11.8 s, T_Cat_ = 78 °C, E= 255.8 mW cm^-2^.

To determine the crystal phase of the support material X-ray diffraction (XRD) measurements have been carried out with a fresh TiO_2_ P25 sample and the thermally reduced Pt/TiO_2_ sample. We used a PANalytical X’Pert Pro MPD (Philips) equipped with a Cu Kα radiation source to collect the XRD patterns. The results and reference patterns for anatase (98-020-2243), rutile (98-006-4987) and platinum (98-006-4924) are displayed in Figure S12.

It can be denoted, that the thermal reduction treatment did not induce any measurable change in crystal phase. The reflexes of both samples are in good agreement with the reference patterns for anatase (major phase) and rutile with no significant Pt reflexes in case of the Pt/TiO_2_ catalyst.

Figure S12: XRD patterns of fresh TiO_2_ P25 and thermally reduced Pt/TiO_2_ with reference patterns for anatase, rutile and platinum.


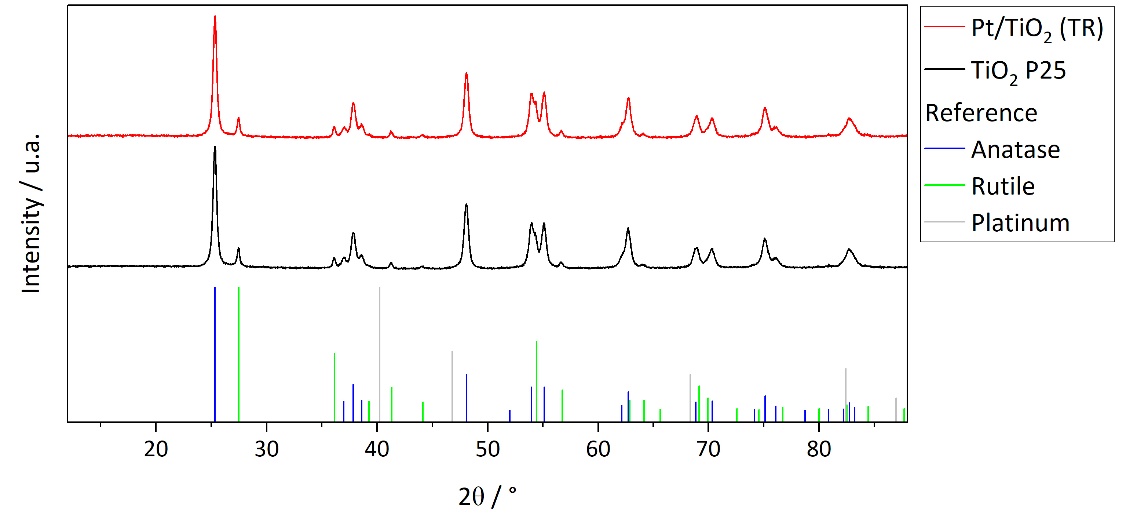


## S9 Oxygen co-dosing - additional data


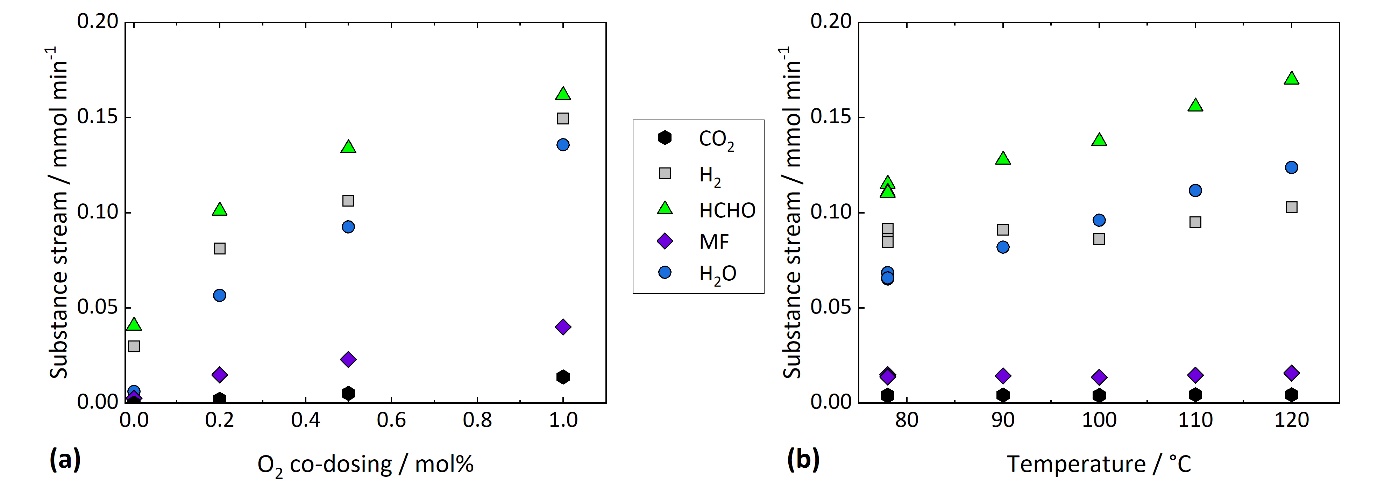


Figure S13: Substance streams at varying oxygen concentrations (a) and temperatures (b) (‘harsh’ activation conditions, no deactivation compensation, E = 255.8 mW cm^‑2^, τ = 11.8 s; (a) T_Cat_ = 78 °C, m_Cat_ = 0.1521 g, ω = 1.12 mg cm^‑2^, thermally reduced; (b) c_O2_ = 0.5 mol%, m_Cat_ = 0.1220 g, ω = 0.90 mg cm^‑2^, thermally reduced).

Figure S14 compares the reaction of MeOH over Pt/TiO_2_ catalyst under dark and UV conditions with and without the influence of O_2_ co-dosing at a fixed temperature of 78 °C and residence time of 23.5 s.


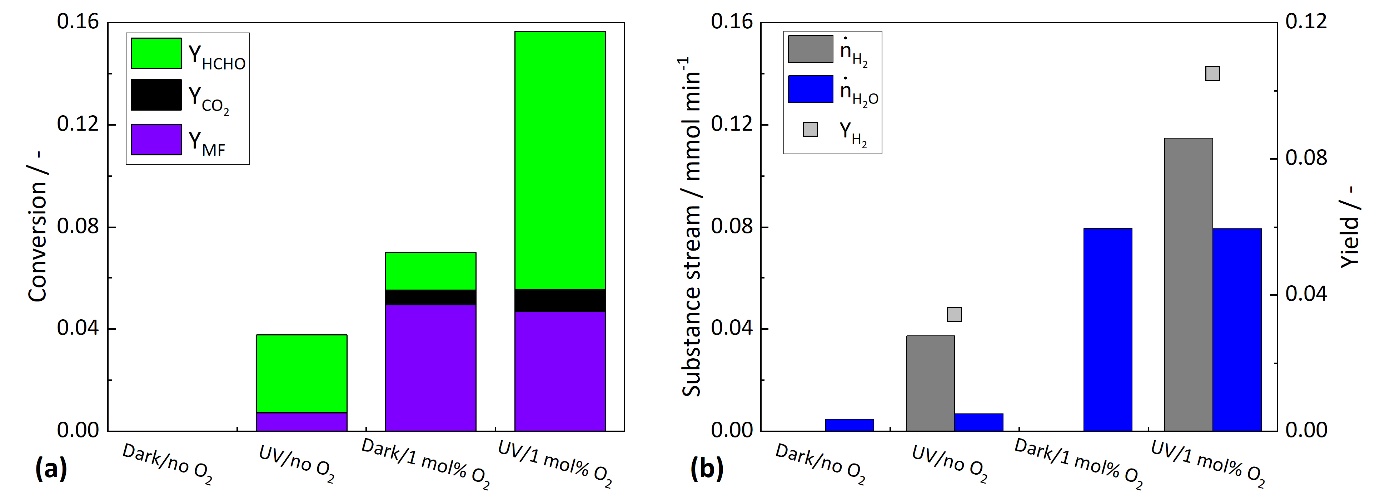


Figure S14: Comparison of dark and UV enhanced reaction of MeOH over Pt/TiO_2_ with and without O_2_ co-dosing. MeOH conversion and yields for HCHO, MF and CO2 (a) as well as H_2_ yield and substance streams of H_2_ and H_2_O (b) (initial ‘harsh’ activation conditions; T = 78 °C, m_Cat_ = 0.1220 g, ω = 0.90 mg cm^-2^, E = 255.8 mW cm^-2^, τ = 23.5 s, thermally reduced).

Without UV light and O_2_ co-dosing no activity is observed. When UV light is switched on a MeOH conversion of 3.8 % is achieved with the main product being HCHO. The low substance stream of water at O_2_ free reaction conditions is a result of impurities in the MeOH feed stream. In contrast to oxygen free operation, using 1 mol% O_2_ co-dosing and dark conditions already about 7.0 % MeOH conversion is achieved. This conversion is attributed to the thermal oxidation of MeOH, because no H_2_ is formed as product and instead significant amounts of water are measured (see Figure S14 (b)). Additionally, the selectivity shifts to MF as main reaction product in this case. Interestingly, the water formation is not increased when the UV light is switched on, while the overall MeOH conversion is further increased to 15.7 %. A clear dependence of H_2_ formation on UV irradiation can be derived from the experiments shown in Figure S14 (b).


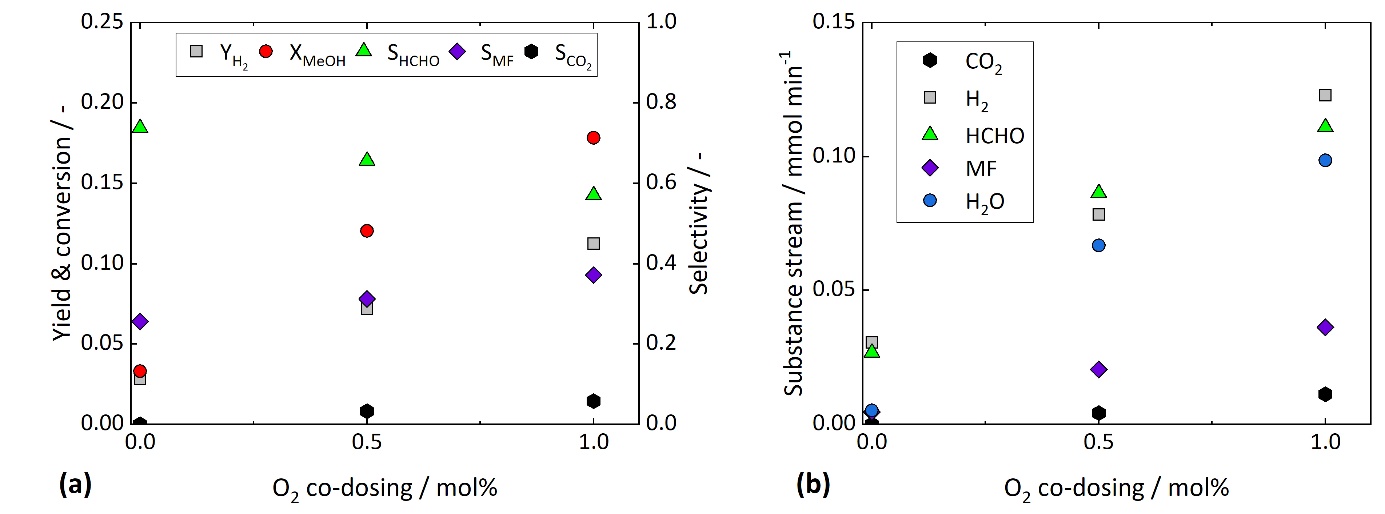


Figure S15: MeOH conversion, H_2_ yield and product selectivities at varying oxygen concentrations (a) and corresponding substance streams (b) (‘harsh’ activation conditions, no deactivation compensation, E = 255.8 mW cm^‑2^, τ= 23.5 s, T_Cat_ = 78 °C, m_Cat_ = 0.1521 g, ω = 1.12 mg cm^‑2^, thermally reduced).


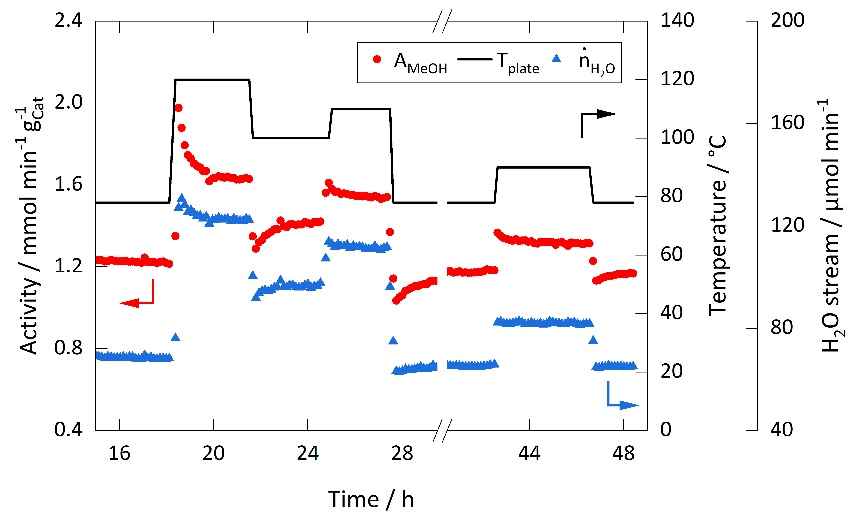


Figure S16: Temperature variation in a continuous flow experiment with temperature range from 78 °C to 120 °C (initial ‘harsh’ activation conditions; c_O2_= 0.5 mol%, m_Cat_ = 0.1220 g, ω = 0.90 mg cm^-2^, E = 255.8 mW cm^-2^, τ = 11.8 s, thermally reduced).
